# Emergence of HPAI H5N6 Clade 2.3.4.4b in Wild Birds: A Case Study From South Korea, 2023

**DOI:** 10.1155/tbed/4141478

**Published:** 2024-11-20

**Authors:** Chang-Gi Jeong, Chung-Young Lee, Su-Beom Chae, Jung-Hoon Kwon, Eun-Jee Na, Jun-Soo Park, Young-Sik Kim, Seung-Chai Kim, Hwan-Ju Kim, Young-Sun Sung, Sun-Young Kim, Won-Il Kim, Jae-Ku Oem

**Affiliations:** ^1^College of Veterinary Medicine, Jeonbuk National University, Iksan 54596, Republic of Korea; ^2^Biosafety Research Institute, Iksan 54596, Republic of Korea; ^3^Department of Microbiology, School of Medicine, Kyungpook National University, 680 Gukchaebosang-ro, Jung-gu, Daegu 41944, Republic of Korea; ^4^College of Veterinary Medicine, Kyungpook National University, 80 Daehak-ro, Daegu 41566, Republic of Korea

## Abstract

The emergence and evolution of avian influenza A viruses (AIVs) pose significant challenges to both public health and animal husbandry worldwide. Here, we characterized a novel reassortant highly pathogenic avian influenza virus (HPAIV), clade 2.3.4.4b H5N6, that was isolated from a mandarin duck in South Korea in December 2023. Phylogenetic and molecular analyses show that the hemagglutinin (HA) gene of the 23-JBN-F12-36/H5N6 virus clustered with HPAIV clade 2.3.4.4b H5N1 viruses, which were circulating in South Korea and Japan in 2022–2023. The M and polymerase acidic (PA) genes also revealed a close association with the HPAIV clade 2.3.4.4b H5N1 AIV that was identified previously in South Korea during November 2022. Notably, the neuraminidase (NA) gene of the 23-JBN-F12-36/H5N6 virus was estimated to have its origins in the HPAIV clade 2.3.4.4h H5N6 prevalent in poultry in China, and it is clustered with the AIVs that are associated with human infection cases. Taken together, these results show that the virus has been produced by reassortment with H5N1 HPAIV, which is prevalent in wild birds; H5N6 HPAIV, which is circulated in poultry in China; and the internal genes of low pathogenic avian influenza viruses (LPAIVs). In light of the reassortment of HPAIVs circulating in existing wild birds and HPAIVs circulating in poultry in China within the 2.3.4.4b H5Nx clade, it is imperative to strengthen active surveillance across wild bird populations, poultry farms, and live poultry markets, and to inform for the effective design of improved prevention and control strategies.

## 1. Introduction

Since the A/goose/Guangdong/1/96 (Gs/GD) lineage was first detected in China in 1996, it has spread worldwide through wild birds, as a result of which it has evolved into different clades (0–9) and subclades based on the hemagglutinin (HA) gene over the last two decades [1]. Most outbreaks of H5 avian influenza A viruses (AIVs) have been geographically confined and successfully eradicated through the implementation of national disease control strategies [2, 3]. Nevertheless, several Gs/GD lineage H5Nx viruses have been spread by migratory birds, which has led to the emergence of the global highly pathogenic avian influenza viruses (HPAIVs) outbreak, and viruses that belong to only clade 2.2, clade 2.3.2, or clade 2.3.4.4 have been maintained through wild birds [4–6]. In particular, clade 2.3.4.4 viruses have emerged globally since 2014, and they have evolved into eight subgroups (2.3.4.4a–2.3.4.4h) according to the phylogenetic classification by the World Health Organization (WHO) [5, 7, 8].

As for HPAIV clade 2.3.4.4b, the HPAIV H5N8 subtype initially dominated, with the first isolation reported in domestic ducks in China [9]. These viruses have been subsequently transmitted to Europe, Asia, and Africa by wild birds, and several new reassortment HPAIVs (H5Nx—including H5N1, H5N3, H5N4, H5N5, and H5N6) have emerged [10–15]. By late 2020, clade 2.3.4.4b H5N8 viruses re-emerged in China, where they originated from wild bird populations [16]. These H5N8 viruses underwent further genetic reassortment with the contemporary H5N6 viruses, ultimately resulting in the emergence of clade 2.3.4.4b H5N6 variants [6, 17]. These H5N6 variants have notably demonstrated a marked host tropism for waterfowl [17].

The specific HPAIV H5N6 strain was first reported in China in 2014, after which it rapidly spread across the country within a few years [18, 19]. As a result of ongoing evolution, the clade 2.3.4.4h H5N6 viruses ascended to dominance in China between 2018 and 2020 [20, 21]. These are mainly prevalent in poultry in China, although they are known to infect other species, including humans [21, 22]. The dominance of the H5N6 virus in China has recently shifted from clade 2.3.4.4h to clade 2.3.4.4b [22]. In a significant development, the transmission of clade 2.3.4.4b H5N6 AIVs across different host species has expanded its host range to include at least six species, notably including humans [22].

The global prevalence of the H5N6 among avian populations, the extensive variety of AIVs currently in circulation, and the frequent interactions among different host species have combined to create a conducive environment for reassortment events and the sustained zoonotic transmission of AIVs [23]. Following the initial reports of H5N6 outbreaks in avian populations across Laos, China, and Vietnam, the first human case of avian-origin H5N6 infection was identified in a poultry dealer from Sichuan Province, China, in 2014. Since then, human cases of H5N6 have been consistently reported each year, with a notable surge in cases occurring in 2021 [24]. Several studies have described that clade 2.3.4.4 H5N6 viruses can bind both avian-origin and human-origin sialic acid (SA) receptors and that they could attach to human tracheal epithelial and alveolar tissues [25], and human infection cases have been reported [23, 24, 26].

In South Korea, the initial detection of two H5N8 AIVs belonging to the clade 2.3.4.4b in wild birds was documented in January 2014; this was a short outbreak and they subsequently disappeared from wild bird populations [27]. Next, there were four HPAI outbreaks, which are shown below with their causative agents: H5N8 in 2016–2017 [28], H5N6 in 2017–2018, and 2018–2019 [29, 30], H5N8 in 2020–2021 [31], and H5N1 and H5N8 in 2021–2022 cocirculation [32], resulting in the loss of a large number of poultry in farms throughout South Korea. To date, there have been no reported cases of human infection with avian influenza within South Korea. However, the intermittent detection of AIVs among both wild and domestic avian populations, combined with the potential threat of viral introduction from abroad via migratory birds, highlights the imperative for continued vigilance and surveillance in the field of influenza research. In the current study, we isolated one novel reassortant HPAIV clade 2.3.4.4b H5N6 from the feces of a mandarin duck collected in South Korea in December 2023 and investigated the evolutionary characteristics of the isolates through whole genome sequencing.

## 2. Materials and Methods

### 2.1. Sample Collection and Virus Isolation

In total, 388 fecal samples of wild birds were collected in December 2023 from a wild bird habitat located in the North Jeolla Province. Fresh and distinctly separated single fecal samples were collected and immediately transported to the laboratory, where they were maintained at a temperature of 4°C. Samples were screened using LiliF AIV M real-time reverse transcription polymerase chain reaction (RT-PCR) Kit (iNtRON Biotechnology, Korea) according to a previously described method [33]. The AIV-positive sample was suspended in 1 × PBS (pH 7.4) containing antibiotics (100 U/*μ* L of penicillin and streptomycin) and centrifuged at 3000 *g* for 10 min. The supernatants were filtered using a sterile 0.45 *μ* m filter (GVS, USA). The filtered supernatants were inoculated into 9–11-day-old specific-pathogen-free embryonated chicken eggs and incubated for 3–4 days at 37°C. Harvested allantoic fluids were extracted by a Miracle-AutoXT Automated Nucleic Acid Extraction System (iNtRON Biotechnology, Korea). AIV subtypes were identified using RT-qPCR by the TOP-realTM one-step RT qPCR Kit (Enzynomics, Korea), as detailed previously [33, 34]. The host of the AIV-positive sample was determined using the mitochondrial cytochrome C oxidase I gene, as described previously [35, 36].

### 2.2. Whole Genome Sequencing

Viral ribonucleic acid (RNA) was extracted from AIV-positive allantoic fluid for multisegment reverse transcription-PCR, as described previously [37]. The PCR amplification was performed using BIONICS index-tagged (BIT) sequencing methods (BIONICS, South Korea), and data on about 100,000 reads were generated. The raw data were assembled using trimmomatic (v0.39) and SPAdes genome assembler (v3.15.4) programs. The nucleotide sequences of the virus were deposited into the EpiFlu database of the Global Initiative for Sharing All Influenza Data (GISAID) (https://gisaid.org/) (Supporting Information 1: Table S1).

### 2.3. Sequence Dataset and Phylogenetic Analysis

The reference HA gene sequences used in the phylogenetic analysis were obtained from the GISAID database. We downloaded all available H5 AIV sequences collected from 2016. We constructed a maximum-likelihood (ML) phylogenetic tree using FastTree v. 2.1.11 [38] and extracted clade 2.3.4.4b viruses that originated from the virus isolated from wild birds in Qinghai/China and Uvs-Nuur Lake/Mongolia in 2016. For effective computation, the number of sequences was reduced based on the sequence identity using the program cluster database at high identity with tolerance (CD-HIT) (v4.8.1) [39]. In total, 835 sequences, including a H5N6 virus isolated in this study, were used for the final phylogenetic tree analysis.

A time-scaled maximum clade credibility (MCC) tree of the HA gene was constructed using BEAST (v.1.10.4). For the estimation of the nucleotide substitution rates, the general time reversible (GTR) model and the uncorrelated lognormal relaxed molecular clock approach were used due to their flexibility. Four independent Markov chain Monte Carlo (MCMC) analyses were performed for the HA segment of AIVs for 50 million generations sampled every 5000 runs. Moreover, the BEAGLE library was utilized to improve computational performance. Tracer (v.1.7.1) was used to evaluate the parameter convergence of MCMC chains. The MCC tree was prepared using TreeAnnotator (v1.10.4) and visualized by FigTree 1.4.2 (http://tree.bio.ed.ac.uk/software/figtree/).

### 2.4. Reassortment Analysis

The full genome sequences for each segment of AIVs and human influenza A viruses isolated during 2018–2024 were obtained from the GISAID database. The HA gene downloaded the H5Nx (H5N1, H5N2, H5N3, etc.) subtype, the neuraminidase (NA) gene obtained HxN6 (H1N6, H2N6, H3N6, etc.), and the remaining six segments collected all subtypes (H1N2, H3N8, H9N2, etc.) (Supporting Information 2: Table S2). The collected sequences were reduced to avoid any unwanted sampling bias for each segment using the CD-HIT program (v4.8.1) [39]. We also downloaded the top 20 sequences with high similarity (top 20 sequences) to our HPAIV isolate. Multiple sequence alignment was conducted with approximately 250 sequences of each segment using Clustal Omega (v.1.2.4). The ML tree was reconstructed using RAxML-NG (v.1.2.0) after finding the best fitting model using ModelFinder of IQ-TREE (v.1.6.12). The branch topology stability within the phylogenetic tree was examined using a comprehensive approach that employed 1000 bootstrap replicates for validation. Constructed ML trees were visualized by the “ggtree” package [40] in R version 4.3.3 (R Core Team, Vienna, Austria) [41].

### 2.5. Evaluation of Amino Acid Substitutions Related to the Adaptation in Mammalian

The alignment of HA sequences was performed using the HA subtype numbering conversion utility, which is available through the Bacterial and Viral Bioinformatics Resource Center (BV-BRC) (https://www.bv-brc.org/). By adopting the H5 numbering scheme for this alignment, we ensured the design of a standardized comparison framework for evaluating the evolutionary dynamics of HA proteins in mammalian species. The other nucleotide sequences were aligned using Clustal Omega (v.1.2.4). The mammalian molecular markers were determined while referring to previous studies [30, 42, 43].

## 3. Results and Discussion

The reassortant HPAIV clade 2.3.4.4 H5N6 viruses emerged in the 2016–2017 and 2017–2018 winter seasons through migratory birds, ultimately leading to large HPAI outbreaks and resulting in the loss of approximately 1 billion birds in 440 poultry farms [30, 44]. Then, in 2020, a previous study reported two novel HPAI clade 2.3.4.4b H5N6 and 10 novel HPAI clade 2.3.4.4c H5N6 viruses [30]. These viruses were identified as being similar to those of viruses isolated in 2016–2018 [30, 45, 46]. Since then, to our knowledge, there have been no reports of H5N6 HPAIVs in Korea.

On December 19, 2023, one H5N6 HPAIV was isolated from the feces of a mandarin duck in Jeongeup-si, South Korea (23-JBN-F12-36). The isolate was identified as the H5N6 subtype and determined as HPAIV including multiple basic amino acids (PLREKRRKR/G) within the cleavage site of the HA gene, which is known to be related to highly pathogenicity phenotype in poultry. Phylogenetic analysis revealed that the 23-JBN-F12-36/H5N6 (sample collection date: December 19, 2023) virus belongs to clade 2.3.4.4b and is almost identical (99.6%–100%) with A/peregrine falcon/Saga/4112A002/H5N6 (EPI_ISL_18740267, sample collection date: December 6, 2023, hereafter “Saga strain”) (Table 1). These two H5N6 HPAIVs are clustered with those of H5N1 HPAIVs that have previously been isolated from wild birds in South Korea and Japan in 2022 (Supporting Information 3: Figure S1). Since these two viruses were isolated at approximately the same time, it is expected that they share a common ancestor. The tMRCA between them was August 7, 2023, suggesting that both viruses likely emerged around August, just before the onset of the major migratory bird influx.

**Figure 1:**
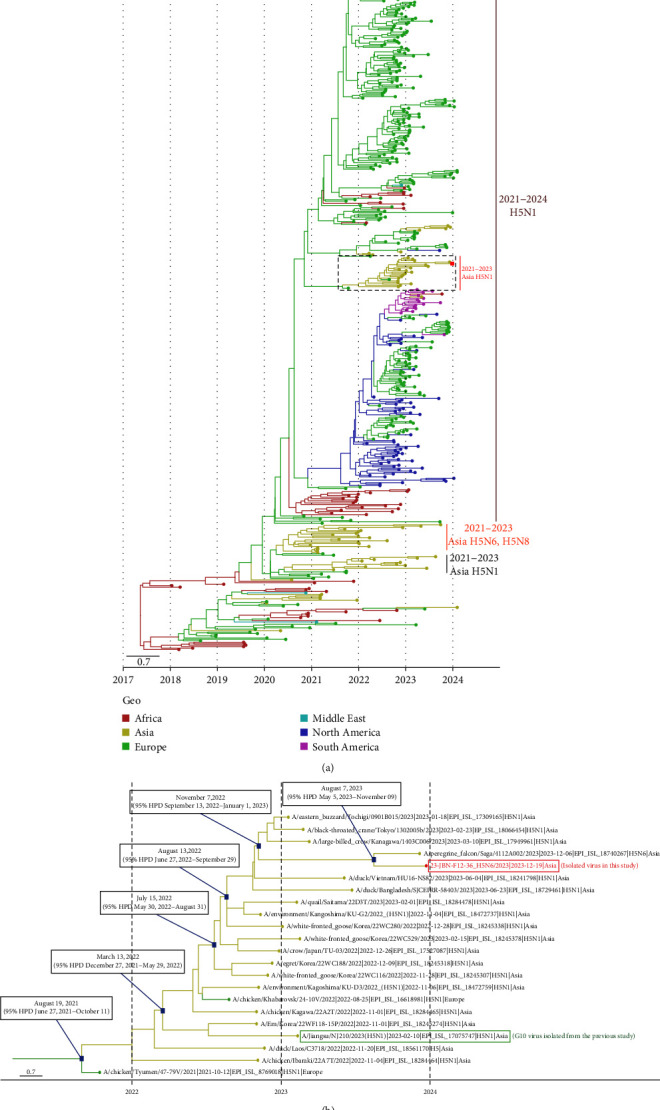
Phylogenetic analysis of novel highly pathogenic avian influenza A (H5) clade 2.3.4.4b virus identified in wild bird feces in South Korea, December 2023. (A) MCC phylogenetic tree of HA gene based on discrete trait analysis of geographic location. The time scale is displayed on the horizontal axis. The color of each branch represents the geographic region. Red squares represent the virus isolated in this study whereas black dotted squares show the group including the isolate. (B) Enlarged view of the black dotted rectangle in (A). The red rectangle and letter indicate novel isolate in South Korea and the green one presents G10 isolate (A/Jiangsu/NJ210/2023 (H5N1/2.3.4.4b)). HA, hemagglutinin; HPD, highest posterior density; MCC, maximum clade credibility.

The polymerase basic 1 (PB1) and M genes were also derived from the clade 2.3.4.4b H5N1 through migratory wild birds between South Korea and Japan. In particular, the HA and M genes were clustered with A/Jiangsu/NJ210/2023 (H5N1/2.3.4.4b, isolated from nasal swab of 53 years old patient), which belongs to genotype G10 (Figure 1 and Supporting Information 3: Figure S1 and Supporting Information 4: Figure S2). These results are similar to those of a previous study indicating that the K22-920 strain isolated from South Korea shared a recent common ancestry with the G10 viruses [43]. Along with these results, in the polymerase acidic (PA) gene of the 23-JBN-F12-36/H5N6, the most closely related virus was A/common teal/Amur region/92b/2020 (H6N2/LPAI) (Supporting Information 4: Figure S2 and Table 1), and the K22-920 strain also possesses the same segment [43]. In addition, the PB1, nucleoprotein (NP), and nonstructural (NS) genes of the K22-920 strain exhibited high similarity (98.4%–99.3%) to low pathogenic avian influenza viruses (LPAIVs) identified in South Korea and Bangladesh during 2019–2020 [43]. Likewise, the 23-JBN-F12-36/H5N6 strain showed a similar reassortment pattern (PB2, NP, and NS) to K22-920. These results indicate a close genetic relationship between the 23-JBN-F12-36/H5N6 strain and K22-920, suggesting that the 23-JBN-F12-36/H5N6 strain likely emerged through additional reassortment events involving the PB2, NP, and NS genes, presumably facilitated by wild birds.

**Figure 2:**
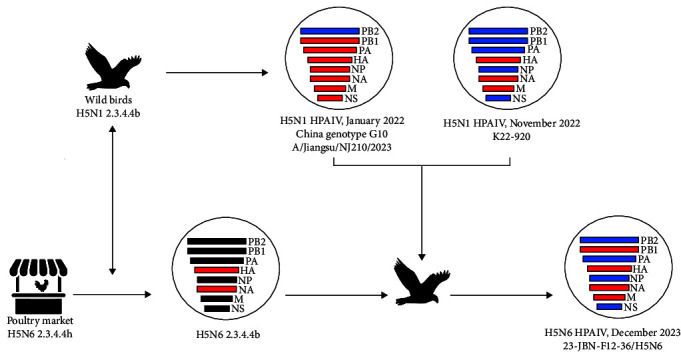
Schematic figure of virus reassortment events. Bars indicate eight gene segments of the AIV. The red bar represents gene segments originating from highly pathogenic AIVs and the blue bar exhibits gene segments originating from low pathogenic AIVs. The black bar means gene segments originating from the AIVs circulating in China. AIV, avian influenza virus.

The NA gene of the 23-JBN-F12-36/H5N6 strain demonstrated phylogenetic clustering with viruses of the clade 2.3.4.4b H5N6, which was isolated in 2021 (Supporting Information 4: Figure S2). Notably, this clade 2.3.4.4b was closely clustered with clade 2.3.4.4h H5N6 viruses identified during the period from 2018 to 2021. This observation suggests that the NA gene of the 23-JBN-F12-36/H5N6 strain likely originated from the clade 2.3.4.4h H5N6. The virus identified in this study also demonstrated the highest sequence homology (98.62%) with A/duck/Hunan/S40199/2021, classified as H5N6/2.3.4.4b HPAIV (Table 1). Moreover, this S40199 H5N6 virus exhibited substantial genetic similarity to clade 2.3.4.4b H5N6 viruses that have previously been isolated from human infection cases (Supporting Information 5: Table S3). The PB2 gene shared high homology with A/environment/Chongqing/1795/2023 (H9N2/LPAIV) from China (Table 1), which was isolated from a live poultry market (Supporting Information 4: Figure S2). Likewise, NS and NP genes are also derived from LPAIVs from East Asia by migratory wild birds. Taken together, the 23-JBN-F12-36/H5N6 virus is hypothesized to have emerged through a series of reassortment events, potentially beginning with the exchange of genetic material between H5N1 viruses circulating in wild avian populations and H5N6 strains prevalent in poultry in China. This may have been followed by additional reassortment involving the internal genes of LPAIVs (Figure 2). However, given the current data, the precise sequence of these reassortment events cannot be definitively determined. It remains possible that the internal gene reassortment involving LPAIVs could have occurred before or concurrently with the H5N1–H5N6 reassortment. The virus is thought to have been introduced into Korea via migratory birds, though the precise timing and locale of these reassortment events (whether in wild birds, poultry farms, or live poultry markets) remain unclear.

The 23-JBN-F12-36/H5N6 virus was found to possess amino acid configurations in the HA that favor binding to avian *α*-2.3-linked SA receptors. Notably, a single amino acid substitution in HA—T156A—was identified, which is associated with an enhanced affinity for human-like receptors (*α*-2.6-linked SA) (Table 2). The virus also exhibited a substitution of L89V in the PB2 gene and mutations N30D and T215A in the M1 protein, alongside P42S and the ESEV motif in the NS gene, which are known markers for heightened virulence in murine models. These findings suggest that the isolate represents a potential zoonotic risk, thus warranting close monitoring for signs of human infection.

## 4. Conclusion

Comprehensive whole genome sequencing and detailed phylogenetic analyses have determined that the 23-JBN-F12-36/H5N6 virus, isolated in December 2023, represents a novel lineage distinct from the Korean H5N6 strains that were identified from 2017 to 2019. The 23-JBN-F12-36/H5N6 virus is postulated to have originated from a reassortment event between H5N1 viruses circulating in wild birds and H5N6 strains prevalent within poultry in China with further reassortment involving the internal genes of LPAIVs. Given the ongoing emergence and worldwide spread of new reassortant clade 2.3.4.4b HPAI H5Nx viruses, this case underscores the need for heightened surveillance in both wild birds and domestic poultry to track the entry, spread, and development of HPAIVs. Additionally, the current study offers new information that is expected to help enhance prevention and control strategies.

## Figures and Tables

**Table 1:** Comparison of nucleotide sequence identities between segments of novel isolate 2.3.4.4b HPAI (H5N6) and in the GISAID Epiflu database.

Segment	Isolate name	Segment ID	Identity (%)
PB2	A/peregrine falcon/Saga/4112A002/2023 (A/H5N6/2.3.4.4b HPAIV)	EPI2898974	99.78
	**A/environment/Chongqing/1795/2023** **(A/H9N2/LPAIV)**	EPI2841012	99.39

PB1	A/peregrine falcon/Saga/4112A002/2023 (A/H5N6/2.3.4.4b HPAI)	EPI2898975	100.00
	**A/common buzzard/Korea/22WC336/2023** **(A/H5N1/2.3.4.4b HPAIV)**	EPI2742993	99.91

PA	A/peregrine falcon/Saga/4112A002/2023 (A/H5N6/2.3.4.4b HPAIV)	EPI2898976	99.91
	**A/common teal/Amur region/92b/2020** **(A/H6N2/LPAIV)**	EPI1849993	99.54

HA	A/peregrine falcon/Saga/4112A002/2023 (A/H5N6/2.3.4.4b HPAIV)	EPI2898977	99.65
	**A/Hooded crane/Korea/22WC215/2022** **(A/H5N1/2.3.4.4b HPAIV)**	EPI2742811	99.53

NP	A/peregrine falcon/Saga/4112A002/2023 (A/H5N6/2.3.4.4b HPAIV)	EPI2898978	99.93
	**A/coot/South Korea/KU-sw-12/2023** **(A/H6N2/LPAIV)**	EPI3001518	98.80

NA	A/peregrine falcon/Saga/4112A002/2023 (A/H5N6/2.3.4.4b HPAIV)	EPI2898979	99.86
	**A/duck/Hunan/S40199/2021** **(A/H5N6/2.3.4.4b HPAIV)**	EPI1997201	98.62

M	A/peregrine falcon/Saga/4112A002/2023 (A/H5N6/2.3.4.4b HPAIV)	EPI2898980	100.00
	**A/mallard/Miyazaki/221121-8/2022** **(A/H5N1/2.3.4.4b HPAIV)**	EPI3077310	99.87

NS	**A/environment/Chongqing/cs2302/2023** **(A/H4N8/LPAIV)**	EPI2782046	99.86
	A/peregrine falcon/Saga/4112A002/2023 (A/H5N6/2.3.4.4b HPAIV)	EPI2898981	99.71

*Note*: Sequences featuring amino acid residues that contribute to greater virulence in mammals are highlighted in bold.

Abbreviations: GISAID, Global Initiative for Sharing All Influenza Data; HA, hemagglutinin; M, matrix; NA, neuraminidase; NP, nucleoprotein; NS, nonstructural; PA, polymerase acidic; PB1, polymerase basic 1; PB2, polymerase basic 2.

**Table 2:** Amino acid substitutions of Korean 2.3.4.4b H5N6 HPAI isolate, December 2023.

23-JBN-F12-36_H5N6	Molecular marker		Remark
HA (H5 numbering)	D94N	D	Related to increased binding to human-like receptor, *α*-2,6 sialic acid [47]
	S123P	R	
	S133A	I	
	N154D	Q	
	**T156A**	**A**	
	T188I	L	
	V210I	P	
	Q222L	N	
	G224S	R	

PB2	**L89V**	**V**	• L89V and D256G mutations in PB2 are known to be associated with increased virulence in mice [48, 49] • Other mutations in PB2 are known to be associated with increased viral replication in mammals [50–52]
	D256G	D	
	Q591K	Q	
	E627K	E	
	D701N	D	

PA	**A515T**	**T**	Associated with transmissibility of H5 in ferrets [53]

NP	Y52NQH	Y	Associated with human BTN3A3 evasion [54]
	F313Y	F	

M1	**N30D**	**D**	Related to increased virulence in mice [55, 56]
	I43M	M	
	**T215A**	**A**	

NS	**P42S**	**S**	Related to increased virulence in mice [57–60]
	80–84 deletion	—	
	L98F	M	
	I101M	D	
	**ESEV**	**ESEV**	

Cleavage site	PLREKRRKR/GLF		Associated with the infectivity and pathogenicity of the virus [61]

*Note:* Sequences featuring amino acid residues that contribute to greater virulence in mammals are given in bold.

Abbreviations: HA, hemagglutinin; NP, nucleoprotein; NS, nonstructural; PA, polymerase acidic; PB2, polymerase basic 2.

## Data Availability

All the sequences used in the current study were deposited in the GISAID (https://www.gisaid.org/), and the list of influenza A viruses is presented in Supporting Information 1: Table S1 and Supporting Information 2: Table S2.

## Supporting Information

Supporting Information 1 Table S1: H5N6 highly pathogenic avian influenza A virus (AIV) isolated in this study.

Supporting Information 2 Table S2: List of influenza A viruses used for maximum-likelihood (ML) tree construction.

Supporting Information 3 Figure S1: ML phylogenetic tree of hemagglutinin (HA) gene segment from influenza A viruses. Branches are colored by clade. The black rectangle represents the virus isolated in the current study. The black rectangle represents the virus isolated in the current study and indicates the G10 isolate (A/Jiangsu/NJ210/2023 (H5N1/2.3.4.4b)). The colored boxes indicate the countries where each virus has been found. A human- and market-shaped pictograms displayed the virus was isolated from humans and the poultry market, respectively.

Supporting Information 4 Figure S2: ML phylogenetic tree of seven segments (polymerase basic 2 [PB2], polymerase basic 1 [PB1], polymerase acidic [PA], nucleoprotein [NP], neuraminidase [NA], matrix [M], and nonstructural [NS]) from influenza A viruses. Branches are colored by subtypes. The black rectangle represents the virus isolated in the current study and indicates the G10 isolate (A/Jiangsu/NJ210/2023 (H5N1/2.3.4.4b)). The colored boxes indicate the countries where each virus has been found. A human-shaped pictogram displayed the virus was isolated from humans.

Supporting Information 5 Table S3: Comparison of NA gene similarity between 23-JBN-F12-36/H5N6 and 20 different strains with high homology.
