# Supplementary material for: Emergence of HPAI H5N6 Clade 2.3.4.4b in Wild Birds: A Case Study From South Korea, 2023
Source: Transbound Emerg Dis. 2024 Nov 20;2024:4141478. doi: 10.1155/tbed/4141478 (PMC12020246; doi:10.1155/tbed/4141478)
Supplement: Supporting Information 3 — Figure S1: ML phylogenetic tree of hemagglutinin (HA) gene segment from influenza A viruses. Branches are colored by clade. The black rectangle represents the virus isolated in the current study. The black rectangle represents the virus isolated in the current study and indicates the G10 isolate (A/Jiangsu/NJ210/2023 (H5N1/2.3.4.4b)). The colored boxes indicate the countries where each virus has been found. A human- and market-shaped pictograms displayed the virus was isolated from humans and the poultry market, respectively. [file 4141478.f3.pdf]

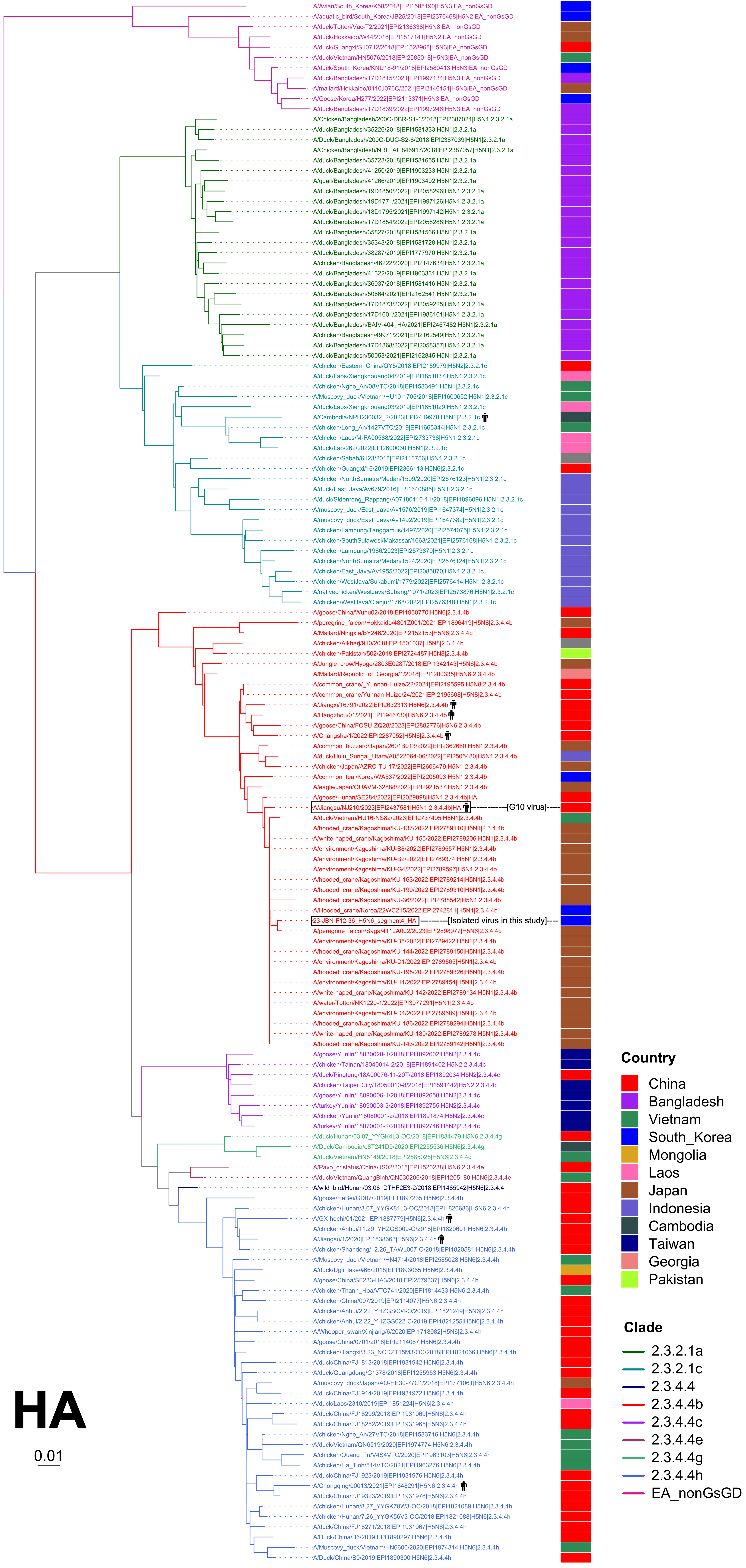
A/Avian/South_Korea/K58/2018|EPI1585190|H5N3|EA_nonGsGD
A/aquatic_bird/South_Korea/JB25/2018|EPI2376468|H5N2|EA_nonGsGD
A/duck/Tottori/Vac-T2/2021|EPI2136338|H5N8|EA_nonGsGD
A/duck/Hokkaido/W44/2018|EPI1817141|H5N2|EA_nonGsGD
A/duck/Guangxi/S10712/2018|EPI1528968|H5N3|EA_nonGsGD
A/duck/Vietnam/HN5076/2018|EPI2585018|H5N3|EA_nonGsGD
A/duck/South_Korea/KNU18-91/2018|EPI2580413|H5N3|EA_nonGsGD
A/duck/Bangladesh/17D1815/2021|EPI1997134|H5N3|EA_nonGsGD
A/mallard/Hokkaido/0110J076C/2021|EPI2146151|H5N3|EA_nonGsGD
A/Goose/Korea/H277/2022|EPI2113371|H5N3|EA_nonGsGD
A/duck/Bangladesh/17D1839/2022|EPI1997246|H5N3|EA_nonGsGD
A/Chicken/Bangladesh/200C-DBR-S1-1/2018|EPI2387024|H5N1|2.3.2.1a
A/duck/Bangladesh/35226/2018|EPI1581333|H5N1|2.3.2.1a
A/Duck/Bangladesh/200O-DUC-S2-8/2018|EPI2387039|H5N1|2.3.2.1a
A/Chicken/Bangladesh/NRL_AI_846917/2018|EPI2387057|H5N1|2.3.2.1a
A/duck/Bangladesh/35723/2018|EPI1581655|H5N1|2.3.2.1a
A/duck/Bangladesh/41250/2019|EPI1903233|H5N1|2.3.2.1a
A/quail/Bangladesh/41266/2019|EPI1903402|H5N1|2.3.2.1a
A/duck/Bangladesh/19D1850/2022|EPI2058296|H5N1|2.3.2.1a
A/duck/Bangladesh/19D1771/2021|EPI1997126|H5N1|2.3.2.1a
A/duck/Bangladesh/18D1795/2021|EPI1997142|H5N1|2.3.2.1a
A/duck/Bangladesh/17D1854/2022|EPI2058288|H5N1|2.3.2.1a
A/duck/Bangladesh/35827/2018|EPI1581566|H5N1|2.3.2.1a
A/duck/Bangladesh/35343/2018|EPI1581728|H5N1|2.3.2.1a
A/duck/Bangladesh/38287/2019|EPI1777970|H5N1|2.3.2.1a
A/chicken/Bangladesh/46222/2020|EPI2147634|H5N1|2.3.2.1a
A/duck/Bangladesh/41322/2019|EPI1903331|H5N1|2.3.2.1a
A/duck/Bangladesh/36037/2018|EPI1581416|H5N1|2.3.2.1a
A/duck/Bangladesh/50664/2021|EPI2162541|H5N1|2.3.2.1a
A/duck/Bangladesh/17D1873/2022|EPI2059225|H5N1|2.3.2.1a
A/duck/Bangladesh/17D1601/2021|EPI1986101|H5N1|2.3.2.1a
A/duck/Bangladesh/BAIV-404_HA/2021|EPI2467482|H5N1|2.3.2.1a
A/chicken/Bangladesh/49971/2021|EPI2162549|H5N1|2.3.2.1a
A/duck/Bangladesh/17D1868/2022|EPI2058357|H5N1|2.3.2.1a
A/duck/Bangladesh/50053/2021|EPI2162845|H5N1|2.3.2.1a
A/chicken/Eastern_China/QY5/2018|EPI2159979|H5N2|2.3.2.1c
A/duck/Laos/Xiengkhouang04/2019|EPI1851037|H5N1|2.3.2.1c
A/chicken/Nghe_An/08VTC/2018|EPI1583491|H5N1|2.3.2.1c
A/Muscovy_duck/Vietnam/HU10-1705/2018|EPI1600652|H5N1|2.3.2.1c
A/duck/Laos/Xiengkhouang03/2019|EPI1851029|H5N1|2.3.2.1c
A/Cambodia/NPH230032_2/2023|EPI2419978|H5N1|2.3.2.1c
A/chicken/Long_An/1427VTC/2019|EPI1665344|H5N1|2.3.2.1c
A/chicken/Laos/M-FA00588/2022|EPI2733738|H5N1|2.3.2.1c
A/duck/Lao/262/2022|EPI2600030|H5N1|2.3.2.1c
A/chicken/Sabah/6123/2018|EPI2116756|H5N1|2.3.2.1c
A/chicken/Guangxi/16/2019|EPI2366113|H5N6|2.3.2.1c
A/chicken/NorthSumatra/Medan/1509/2020|EPI2576123|H5N1|2.3.2.1c
A/duck/East_Java/Av679/2016|EPI1640885|H5N1|2.3.2.1c
A/duck/Sidenreng_Rappang/A07180110-11/2018|EPI1896096|H5N1|2.3.2.1c
A/muscovy_duck/East_Java/Av1576/2019|EPI1647374|H5N1|2.3.2.1c
A/muscovy_duck/East_Java/Av1492/2019|EPI1647382|H5N1|2.3.2.1c
A/chicken/Lampung/Tanggamus/1497/2020|EPI2574075|H5N1|2.3.2.1c
A/chicken/SouthSulawesi/Makassar/1663/2021|EPI2576168|H5N1|2.3.2.1c
A/chicken/Lampung/1986/2023|EPI2573879|H5N1|2.3.2.1c
A/chicken/NorthSumatra/Medan/1524/2020|EPI2576124|H5N1|2.3.2.1c
A/chicken/East_Java/Av1955/2022|EPI2085870|H5N1|2.3.2.1c
A/chicken/WestJava/Sukabumi/1779/2022|EPI2576414|H5N1|2.3.2.1c
A/nativechicken/WestJava/Subang/1971/2023|EPI2573876|H5N1|2.3.2.1c
A/chicken/WestJava/Cianjur/1768/2022|EPI2576348|H5N1|2.3.2.1c
A/goose/China/Wuhu02/2018|EPI1930770|H5N6|2.3.4.4b
A/peregrine_falcon/Hokkaido/4801Z001/2021|EPI1896419|H5N8|2.3.4.4b
A/Mallard/Ningxia/BY246/2020|EPI2152153|H5N8|2.3.4.4b
A/chicken/Alkharj/910/2018|EPI1501037|H5N8|2.3.4.4b
A/chicken/Pakistan/502/2018|EPI2724487|H5N8|2.3.4.4b
A/Jungle_crow/Hyogo/2803E028T/2018|EPI1342143|H5N6|2.3.4.4b
A/Mallard/Republic_of_Georgia/1/2018|EPI1200335|H5N6|2.3.4.4b
A/common_crane/_Yunnan-Huize/22/2021|EPI2195595|H5N8|2.3.4.4b
A/common_crane/Yunnan-Huize/24/2021|EPI2195608|H5N8|2.3.4.4b
A/Jiangxi/16791/2022|EPI2632313|H5N6|2.3.4.4b
A/Hangzhou/01/2021|EPI1946730|H5N6|2.3.4.4b
A/goose/China/FOSU-ZQ28/2023|EPI2882776|H5N6|2.3.4.4b
A/Changsha/1/2022|EPI2287052|H5N6|2.3.4.4b
A/common_buzzard/Japan/2601B013/2022|EPI2362660|H5N1|2.3.4.4b
A/duck/Hulu_Sungai_Utara/A0522064-06/2022|EPI2505480|H5N1|2.3.4.4b
A/chicken/Japan/AZRC-TU-17/2022|EPI2606479|H5N1|2.3.4.4b
A/common_teal/Korea/WA537/2022|EPI2205093|H5N1|2.3.4.4b
A/eagle/Japan/OUAVM-62888/2022|EPI2921537|H5N1|2.3.4.4b
A/goose/Hunan/SE284/2022|EPI2029898|H5N1|2.3.4.4b|HA
A/Jiangsu/NJ210/2023|EPI2437581|H5N1|2.3.4.4b|HA -------------[G10 virus]-----
A/duck/Vietnam/HU16-NS82/2023|EPI2737495|H5N1|2.3.4.4b
A/hooded_crane/Kagoshima/KU-137/2022|EPI2789110|H5N1|2.3.4.4b
A/white-naped_crane/Kagoshima/KU-155/2022|EPI2789206|H5N1|2.3.4.4b
A/environment/Kagoshima/KU-B8/2022|EPI2789557|H5N1|2.3.4.4b
A/environment/Kagoshima/KU-B2/2022|EPI2789374|H5N1|2.3.4.4b
A/environment/Kagoshima/KU-G4/2022|EPI2789597|H5N1|2.3.4.4b
A/hooded_crane/Kagoshima/KU-163/2022|EPI2789214|H5N1|2.3.4.4b
A/hooded_crane/Kagoshima/KU-190/2022|EPI2789310|H5N1|2.3.4.4b
A/hooded_crane/Kagoshima/KU-36/2022|EPI2788542|H5N1|2.3.4.4b
A/Hooded_crane/Korea/22WC215/2022|EPI2742811|H5N1|2.3.4.4b
23-JBN-F12-36_H5N6_segment4_HA ----------[Isolated virus in this study]----
A/peregrine_falcon/Saga/4112A002/2023|EPI2898977|H5N6|2.3.4.4b
A/environment/Kagoshima/KU-B5/2022|EPI2789422|H5N1|2.3.4.4b
A/hooded_crane/Kagoshima/KU-144/2022|EPI2789150|H5N1|2.3.4.4b
A/environment/Kagoshima/KU-D1/2022|EPI2789565|H5N1|2.3.4.4b
A/hooded_crane/Kagoshima/KU-195/2022|EPI2789326|H5N1|2.3.4.4b
A/environment/Kagoshima/KU-H1/2022|EPI2789454|H5N1|2.3.4.4b
A/white-naped_crane/Kagoshima/KU-142/2022|EPI2789134|H5N1|2.3.4.4b
A/water/Tottori/NK1220-1/2022|EPI3077291|H5N1|2.3.4.4b
A/environment/Kagoshima/KU-D4/2022|EPI2789589|H5N1|2.3.4.4b
A/hooded_crane/Kagoshima/KU-186/2022|EPI2789294|H5N1|2.3.4.4b
A/white-naped_crane/Kagoshima/KU-180/2022|EPI2789278|H5N1|2.3.4.4b
A/hooded_crane/Kagoshima/KU-143/2022|EPI2789142|H5N1|2.3.4.4b
A/goose/Yunlin/18030020-1/2018|EPI1892602|H5N2|2.3.4.4c
A/chicken/Tainan/18040014-2/2018|EPI1891402|H5N2|2.3.4.4c
A/duck/Pingtung/18A00076-11-20T/2018|EPI1892034|H5N2|2.3.4.4c
A/chicken/Taipei_City/18050010-8/2018|EPI1891442|H5N2|2.3.4.4c
A/goose/Yunlin/18090006-1/2018|EPI1892658|H5N2|2.3.4.4c
A/turkey/Yunlin/18090003-3/2018|EPI1892755|H5N2|2.3.4.4c
A/chicken/Yunlin/18060001-2/2018|EPI1891874|H5N2|2.3.4.4c
A/turkey/Yunlin/18070001-2/2018|EPI1892746|H5N2|2.3.4.4c
A/duck/Hunan/03.07_YYGK4L3-OC/2018|EPI1834479|H5N6|2.3.4.4g
A/Duck/Cambodia/e8T241D9/2020|EPI2255536|H5N6|2.3.4.4g
A/duck/Vietnam/HN5149/2018|EPI2585025|H5N6|2.3.4.4g
A/Pavo_cristatus/China/JS02/2018|EPI1520238|H5N6|2.3.4.4e
A/duck/Vietnam/QuangBinh/QN530206/2018|EPI1205180|H5N6|2.3.4.4e
A/wild_bird/Hunan/03.08_DTHF2E3-2/2018|EPI1485942|H5N6|2.3.4.4
A/goose/HeBei/GD07/2019|EPI1897235|H5N6|2.3.4.4h
A/chicken/Hunan/3.07_YYGK81L3-OC/2018|EPI1820686|H5N6|2.3.4.4h
A/GX-hechi/01/2021|EPI1887779|H5N6|2.3.4.4h
A/chicken/Anhui/11.29_YHZGS009-O/2018|EPI1820601|H5N6|2.3.4.4h
A/Jiangsu/1/2020|EPI1838663|H5N6|2.3.4.4h
A/chicken/Shandong/12.26_TAWL007-O/2018|EPI1820581|H5N6|2.3.4.4h
A/Muscovy_duck/Vietnam/HN4714/2018|EPI2585028|H5N6|2.3.4.4h
A/duck/Ugii_lake/#66/2018|EPI1893065|H5N6|2.3.4.4h
A/goose/China/SF233-HA3/2018|EPI2579337|H5N6|2.3.4.4h
A/chicken/Thanh_Hoa/VTC741/2020|EPI1814433|H5N6|2.3.4.4h
A/chicken/China/007/2019|EPI2114077|H5N6|2.3.4.4h
A/chicken/Anhui/2.22_YHZGS004-O/2019|EPI1821249|H5N6|2.3.4.4h
A/chicken/Anhui/2.22_YHZGS022-C/2019|EPI1821255|H5N6|2.3.4.4h
A/Whooper_swan/Xinjiang/6/2020|EPI1718982|H5N6|2.3.4.4h
A/goose/China/0701/2018|EPI2114087|H5N6|2.3.4.4h
A/chicken/Jiangxi/3.23_NCDZT15M3-OC/2018|EPI1821066|H5N6|2.3.4.4h
A/duck/China/FJ1813/2018|EPI1931942|H5N6|2.3.4.4h
A/duck/Guangdong/G1378/2018|EPI1255953|H5N6|2.3.4.4h
A/muscovy_duck/Japan/AQ-HE30-77C1/2018|EPI1771061|H5N6|2.3.4.4h
A/duck/China/FJ1914/2019|EPI1931972|H5N6|2.3.4.4h
A/duck/Laos/2310/2019|EPI1851224|H5N6|2.3.4.4h
A/duck/China/FJ18299/2018|EPI1931969|H5N6|2.3.4.4h
A/duck/China/FJ18252/2019|EPI1931965|H5N6|2.3.4.4h
A/chicken/Nghe_An/27VTC/2018|EPI1583716|H5N6|2.3.4.4h
A/duck/Vietnam/QN6519/2020|EPI1974774|H5N6|2.3.4.4h
A/chicken/Quang_Tri/V4S4VTC/2020|EPI1963103|H5N6|2.3.4.4h
A/chicken/Ha_Tinh/514VTC/2021|EPI1963276|H5N6|2.3.4.4h
A/duck/China/FJ1923/2019|EPI1931976|H5N6|2.3.4.4h
A/Chongqing/00013/2021|EPI1848291|H5N6|2.3.4.4h
A/duck/China/FJ19323/2019|EPI1931978|H5N6|2.3.4.4h
A/chicken/Hunan/8.27_YYGK70W3-OC/2018|EPI1821089|H5N6|2.3.4.4h
A/chicken/Hunan/7.26_YYGK56V3-OC/2018|EPI1821088|H5N6|2.3.4.4h
A/duck/China/FJ18271/2018|EPI1931967|H5N6|2.3.4.4h
A/Duck/China/B6/2019|EPI1890297|H5N6|2.3.4.4h
A/Muscovy_duck/Vietnam/HN6606/2020|EPI1974314|H5N6|2.3.4.4h
A/Duck/China/B9/2019|EPI1890300|H5N6|2.3.4.4h
Country
China
Bangladesh
Vietnam
South_Korea
Mongolia
Laos
Japan
Indonesia
Cambodia
Taiwan
Georgia
Pakistan
Clade
2.3.2.1a
2.3.2.1c
2.3.4.4
2.3.4.4b
2.3.4.4c
2.3.4.4e
2.3.4.4g
2.3.4.4h
EA_nonGsGD
HA
0.01
