# Supplementary material for: Emergence of HPAI H5N6 Clade 2.3.4.4b in Wild Birds: A Case Study From South Korea, 2023
Source: Transbound Emerg Dis. 2024 Nov 20;2024:4141478. doi: 10.1155/tbed/4141478 (PMC12020246; doi:10.1155/tbed/4141478)
Supplement: Supporting Information 4 — Figure S2: ML phylogenetic tree of seven segments (polymerase basic 2 [PB2], polymerase basic 1 [PB1], polymerase acidic [PA], nucleoprotein [NP], neuraminidase [NA], matrix [M], and nonstructural [NS]) from influenza A viruses. Branches are colored by subtypes. The black rectangle represents the virus isolated in the current study and indicates the G10 isolate (A/Jiangsu/NJ210/2023 (H5N1/2.3.4.4b)). The colored boxes indicate the countries where each virus has been found. A human-shaped pictogram displayed the virus was isolated from humans. [file 4141478.f4.pdf]

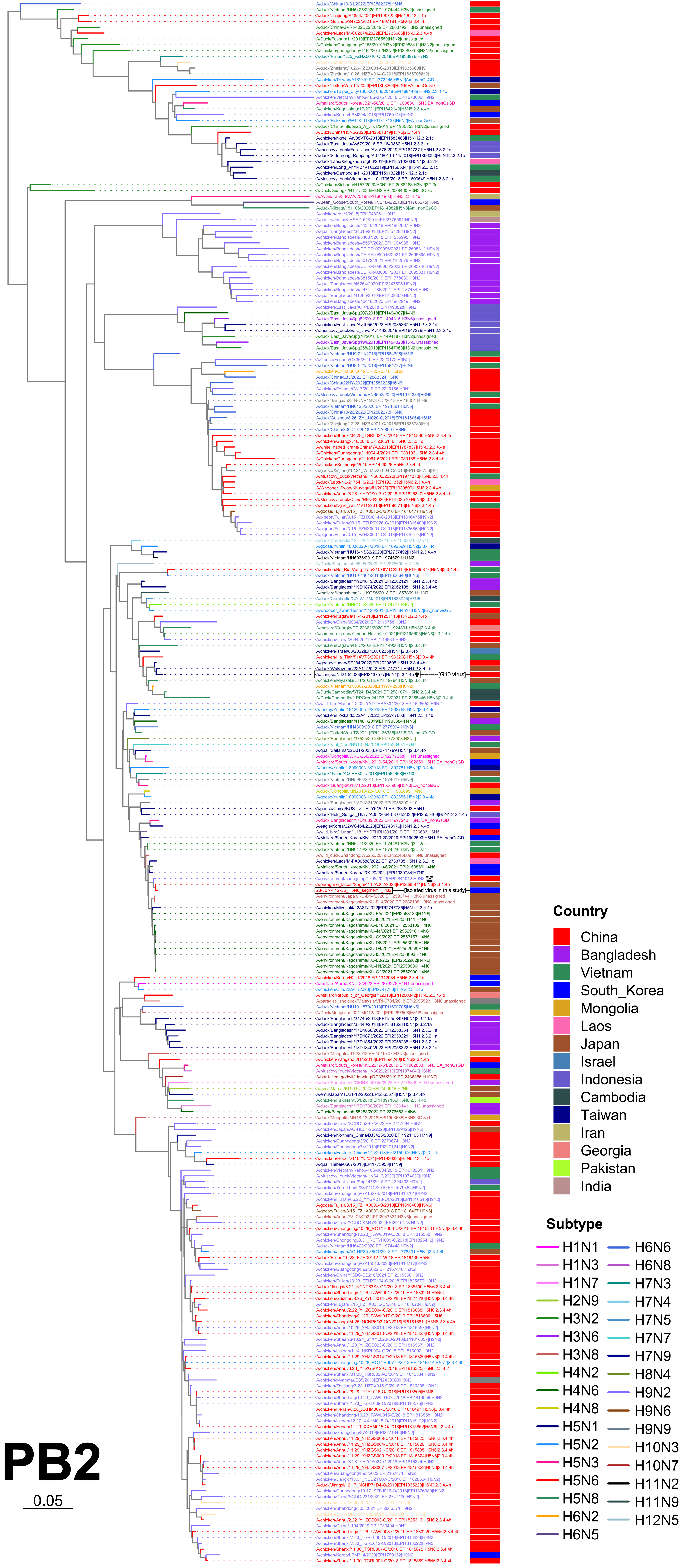

[G10 virus]
[Isolated virus in this study]
Country
China
Bangladesh
Vietnam
South_Korea
Mongolia
Laos
Japan
Israel
Indonesia
Cambodia
Taiwan
Iran
Georgia
Pakistan
India
Subtype
H1N1
H1N3
H1N7
H2N9
H3N2
H3N6
H3N8
H4N2
H4N6
H4N8
H5N1
H5N2
H5N3
H5N6
H5N8
H6N2
H6N5
H6N6
H6N8
H7N3
H7N4
H7N5
H7N7
H7N9
H8N4
H9N2
H9N6
H9N9
H10N3
H10N7
H11N2
H11N9
H12N5
PB2
0.05

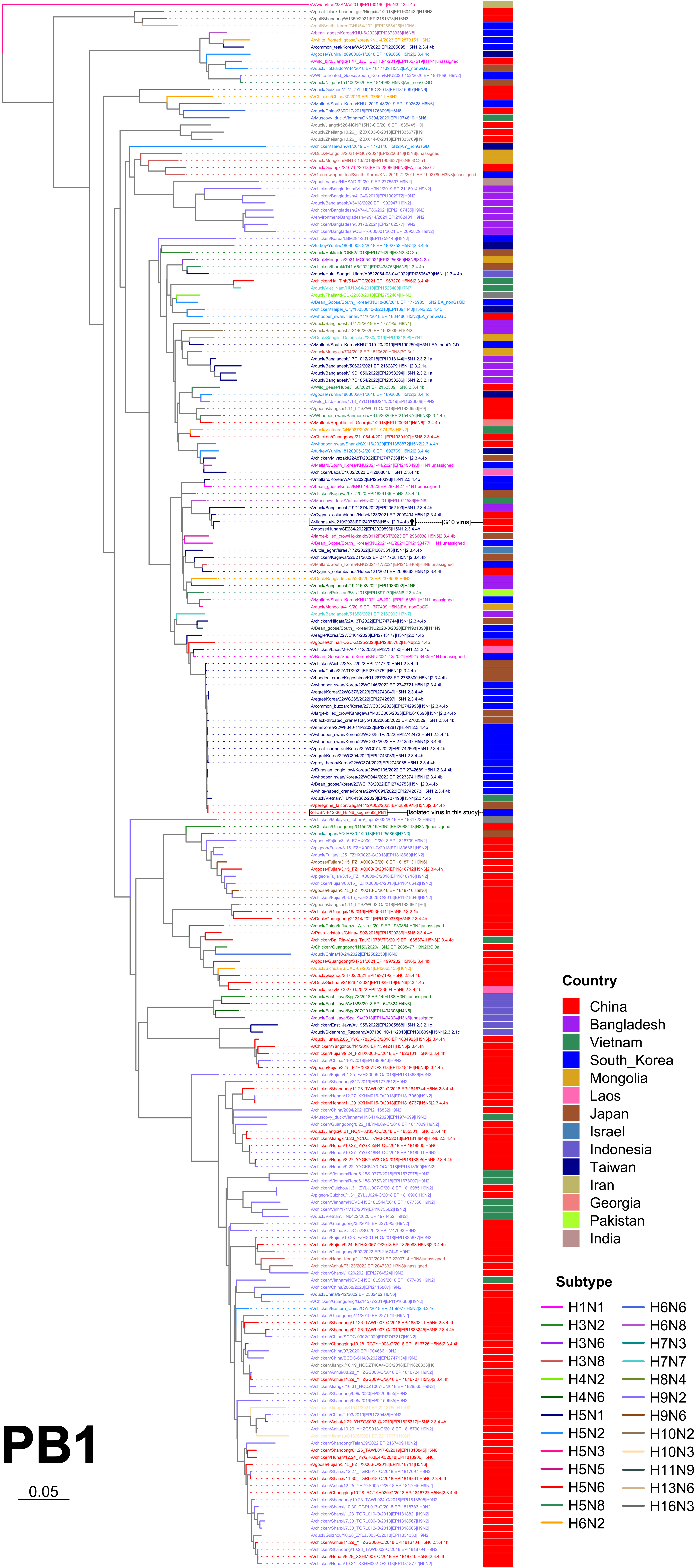
[G10 virus]
[Isolated virus in this study]
Country
China
Bangladesh
Vietnam
South_Korea
Mongolia
Laos
Japan
Israel
Indonesia
Taiwan
Iran
Georgia
Pakistan
India
Subtype
H1N1
H3N2
H3N6
H3N8
H4N2
H4N6
H5N1
H5N2
H5N3
H5N5
H5N6
H5N8
H6N2
H6N6
H6N8
H7N3
H7N7
H8N4
H9N2
H9N6
H10N2
H10N3
H11N9
H13N6
H16N3
PB1
0.05

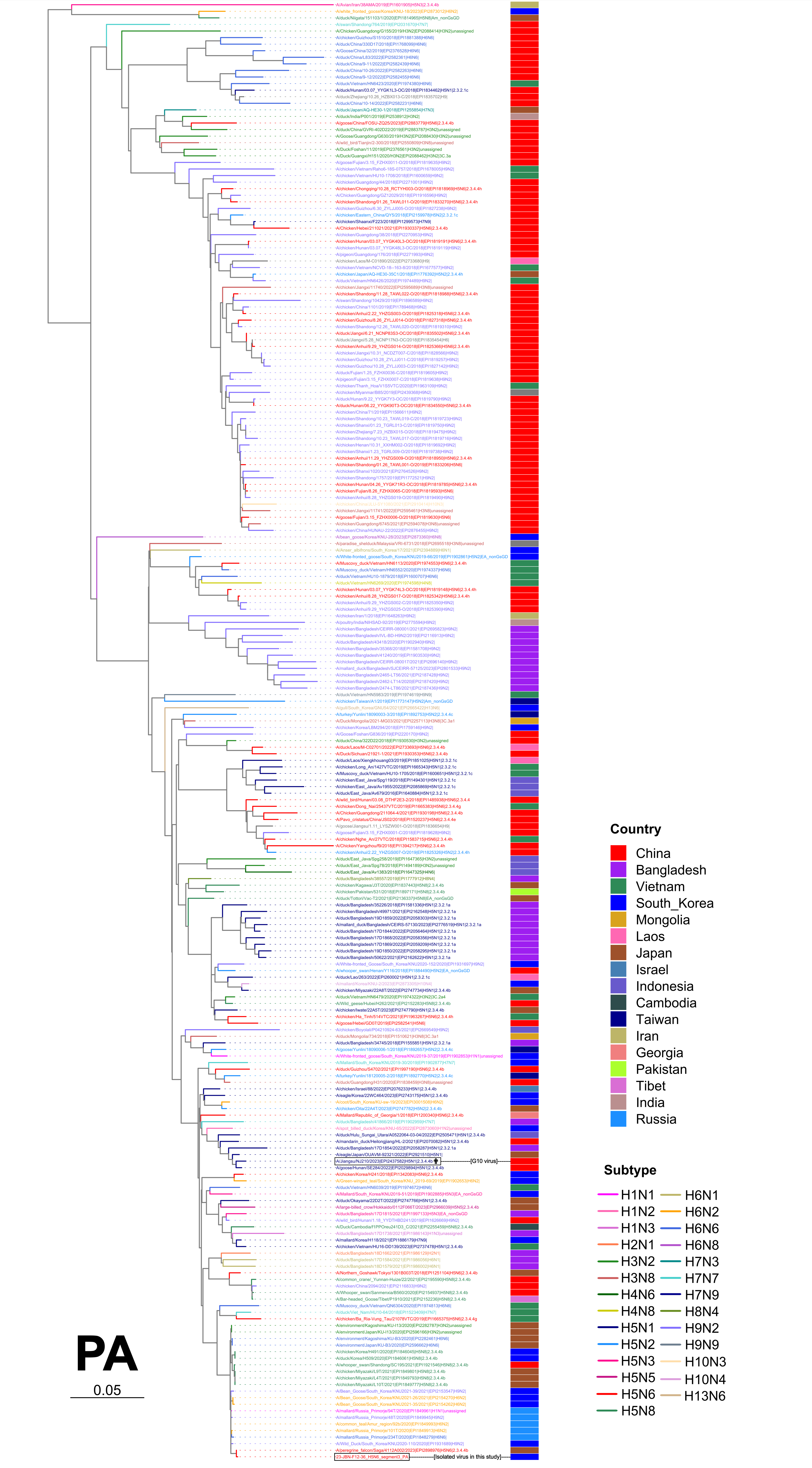

PA
0.05
Country
China
Bangladesh
Vietnam
South_Korea
Mongolia
Laos
Japan
Israel
Indonesia
Cambodia
Taiwan
Iran
Georgia
Pakistan
Tibet
India
Russia
Subtype
H1N1
H1N2
H1N3
H2N1
H3N2
H3N8
H4N6
H4N8
H5N1
H5N2
H5N3
H5N5
H5N6
H5N8
H6N1
H6N2
H6N6
H6N8
H7N3
H7N7
H7N9
H8N4
H9N2
H9N9
H10N3
H10N4
H13N6
[G10 virus]
[Isolated virus in this study]

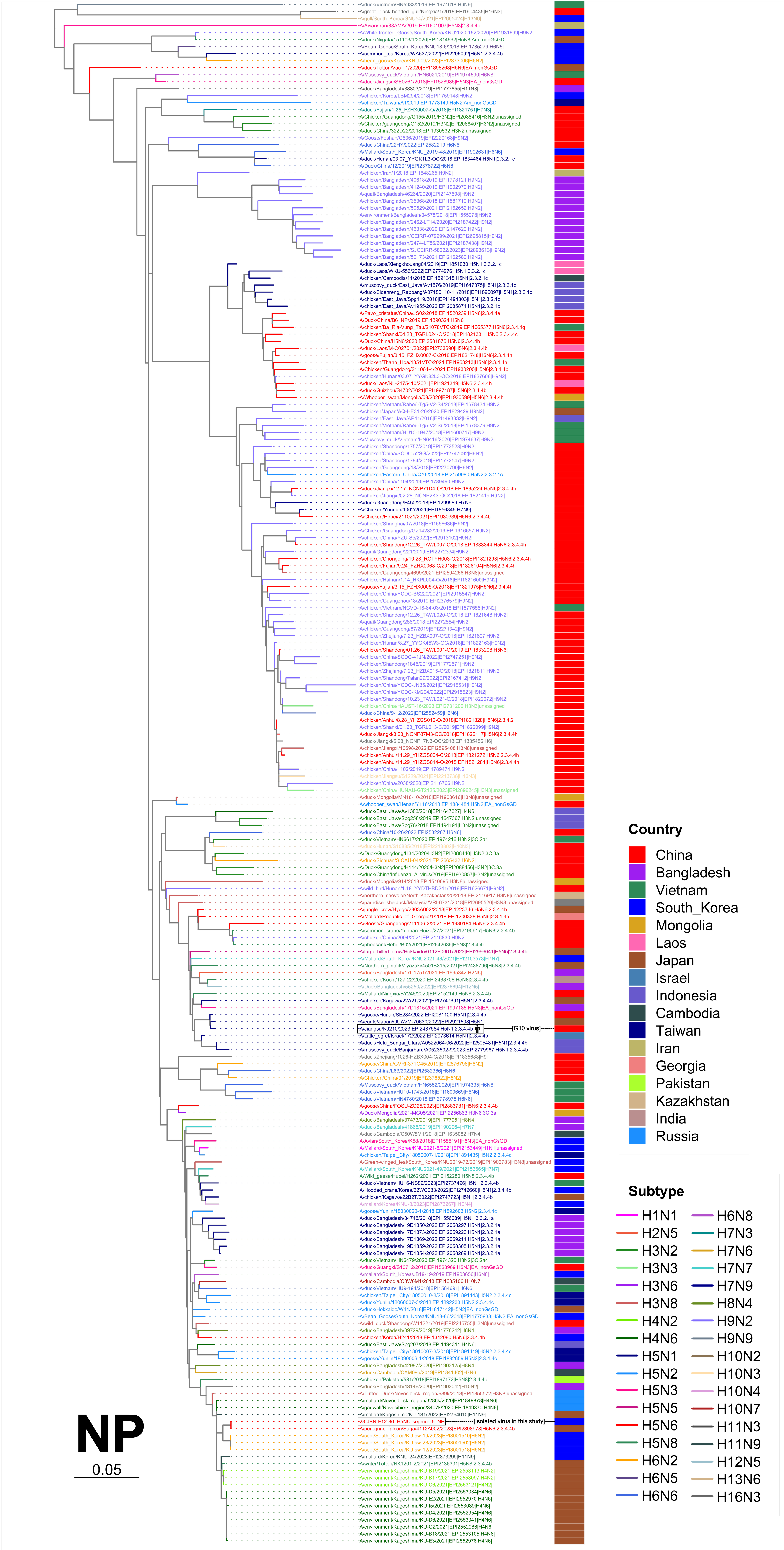

NP
0.05
[G10 virus]
[Isolated virus in this study]
Country
China
Bangladesh
Vietnam
South_Korea
Mongolia
Laos
Japan
Israel
Indonesia
Cambodia
Taiwan
Iran
Georgia
Pakistan
Kazakhstan
India
Russia
Subtype
H1N1
H2N5
H3N2
H3N3
H3N6
H3N8
H4N2
H4N6
H5N1
H5N2
H5N3
H5N5
H5N6
H5N8
H6N2
H6N5
H6N6
H6N8
H7N3
H7N6
H7N7
H7N9
H8N4
H9N2
H9N9
H10N2
H10N3
H10N4
H10N7
H11N3
H11N9
H12N5
H13N6
H16N3

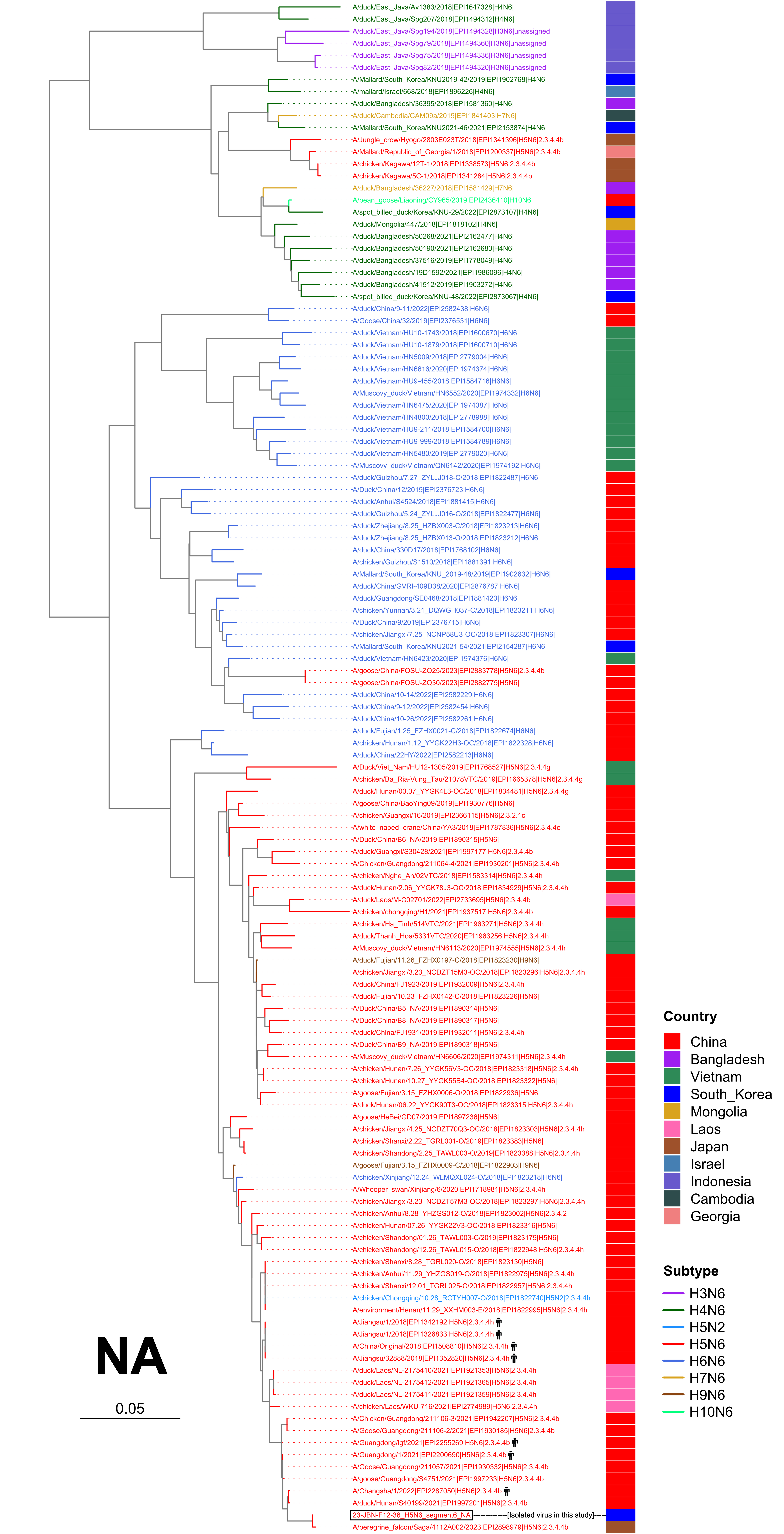

A/duck/East_Java/Av1383/2018|EPI1647328|H4N6|
A/duck/East_Java/Spg207/2018|EPI1494312|H4N6|
A/duck/East_Java/Spg194/2018|EPI1494328|H3N6|unassigned
A/duck/East_Java/Spg79/2018|EPI1494360|H3N6|unassigned
A/duck/East_Java/Spg75/2018|EPI1494336|H3N6|unassigned
A/duck/East_Java/Spg82/2018|EPI1494320|H3N6|unassigned
A/Mallard/South_Korea/KNU2019-42/2019|EPI1902768|H4N6|
A/mallard/Israel/668/2018|EPI1896226|H4N6|
A/duck/Bangladesh/36395/2018|EPI1581360|H4N6|
A/duck/Cambodia/CAM09a/2019|EPI1841403|H7N6|
A/Mallard/South_Korea/KNU2021-46/2021|EPI2153874|H4N6|
A/Jungle_crow/Hyogo/2803E023T/2018|EPI1341396|H5N6|2.3.4.4b
A/Mallard/Republic_of_Georgia/1/2018|EPI1200337|H5N6|2.3.4.4b
A/chicken/Kagawa/12T-1/2018|EPI1338573|H5N6|2.3.4.4b
A/chicken/Kagawa/5C-1/2018|EPI1341284|H5N6|2.3.4.4b
A/duck/Bangladesh/36227/2018|EPI1581429|H7N6|
A/bean_goose/Liaoning/CY965/2019|EPI2436410|H10N6|
A/spot_billed_duck/Korea/KNU-29/2022|EPI2873107|H4N6|
A/duck/Mongolia/447/2018|EPI1818102|H4N6|
A/duck/Bangladesh/50268/2021|EPI2162477|H4N6|
A/duck/Bangladesh/50190/2021|EPI2162683|H4N6|
A/duck/Bangladesh/37516/2019|EPI1778049|H4N6|
A/duck/Bangladesh/19D1592/2021|EPI1986096|H4N6|
A/duck/Bangladesh/41512/2019|EPI1903272|H4N6|
A/spot_billed_duck/Korea/KNU-48/2022|EPI2873067|H4N6|
A/duck/China/9-11/2022|EPI2582438|H6N6|
A/Goose/China/32/2019|EPI2376531|H6N6|
A/duck/Vietnam/HU10-1743/2018|EPI1600670|H6N6|
A/duck/Vietnam/HU10-1879/2018|EPI1600710|H6N6|
A/duck/Vietnam/HN5009/2018|EPI2779004|H6N6|
A/duck/Vietnam/HN6616/2020|EPI1974374|H6N6|
A/duck/Vietnam/HU9-455/2018|EPI1584716|H6N6|
A/Muscovy_duck/Vietnam/HN6552/2020|EPI1974332|H6N6|
A/duck/Vietnam/HN6475/2020|EPI1974387|H6N6|
A/duck/Vietnam/HN4800/2018|EPI2778988|H6N6|
A/duck/Vietnam/HU9-211/2018|EPI1584700|H6N6|
A/duck/Vietnam/HU9-999/2018|EPI1584789|H6N6|
A/duck/Vietnam/HN5480/2019|EPI2779020|H6N6|
A/Muscovy_duck/Vietnam/QN6142/2020|EPI1974192|H6N6|
A/duck/Guizhou/7.27_ZYLJJ018-C/2018|EPI1822487|H6N6|
A/Duck/China/12/2019|EPI2376723|H6N6|
A/duck/Anhui/S4524/2018|EPI1881415|H6N6|
A/duck/Guizhou/5.24_ZYLJJ016-O/2018|EPI1822477|H6N6|
A/duck/Zhejiang/8.25_HZBX003-C/2018|EPI1823213|H6N6|
A/duck/Zhejiang/8.25_HZBX013-O/2018|EPI1823212|H6N6|
A/duck/China/330D17/2018|EPI1768102|H6N6|
A/chicken/Guizhou/S1510/2018|EPI1881391|H6N6|
A/Mallard/South_Korea/KNU_2019-48/2019|EPI1902632|H6N6|
A/duck/China/GVRI-409D38/2020|EPI2876787|H6N6|
A/duck/Guangdong/SE0468/2018|EPI1881423|H6N6|
A/chicken/Yunnan/3.21_DQWGH037-C/2018|EPI1823211|H6N6|
A/Duck/China/9/2019|EPI2376715|H6N6|
A/chicken/Jiangxi/7.25_NCNP58U3-OC/2018|EPI1823307|H6N6|
A/Mallard/South_Korea/KNU2021-54/2021|EPI2154287|H6N6|
A/duck/Vietnam/HN6423/2020|EPI1974376|H6N6|
A/goose/China/FOSU-ZQ25/2023|EPI2883778|H5N6|2.3.4.4b
A/goose/China/FOSU-ZQ30/2023|EPI2882775|H5N6|
A/duck/China/10-14/2022|EPI2582229|H6N6|
A/duck/China/9-12/2022|EPI2582454|H6N6|
A/duck/China/10-26/2022|EPI2582261|H6N6|
A/duck/Fujian/1.25_FZHX0021-C/2018|EPI1822674|H6N6|
A/chicken/Hunan/1.12_YYGK22H3-OC/2018|EPI1822328|H6N6|
A/duck/China/22HY/2022|EPI2582213|H6N6|
A/Duck/Viet_Nam/HU12-1305/2019|EPI1768527|H5N6|2.3.4.4g
A/chicken/Ba_Ria-Vung_Tau/21078VTC/2019|EPI1665378|H5N6|2.3.4.4g
A/duck/Hunan/03.07_YYGK4L3-OC/2018|EPI1834481|H5N6|2.3.4.4g
A/goose/China/BaoYing09/2019|EPI1930776|H5N6|
A/chicken/Guangxi/16/2019|EPI2366115|H5N6|2.3.2.1c
A/white_naped_crane/China/YA3/2018|EPI1787836|H5N6|2.3.4.4e
A/Duck/China/B6_NA/2019|EPI1890315|H5N6|
A/duck/Guangxi/S30428/2021|EPI1997177|H5N6|2.3.4.4b
A/Chicken/Guangdong/211064-4/2021|EPI1930201|H5N6|2.3.4.4b
A/chicken/Nghe_An/02VTC/2018|EPI1583314|H5N6|2.3.4.4h
A/duck/Hunan/2.06_YYGK78J3-OC/2018|EPI1834929|H5N6|2.3.4.4h
A/duck/Laos/M-C02701/2022|EPI2733695|H5N6|2.3.4.4b
A/chicken/chongqing/H1/2021|EPI1937517|H5N6|2.3.4.4b
A/chicken/Ha_Tinh/514VTC/2021|EPI1963271|H5N6|2.3.4.4h
A/duck/Thanh_Hoa/5331VTC/2020|EPI1963256|H5N6|2.3.4.4h
A/Muscovy_duck/Vietnam/HN6113/2020|EPI1974555|H5N6|2.3.4.4h
A/duck/Fujian/11.26_FZHX0197-C/2018|EPI1823230|H9N6|
A/chicken/Jiangxi/3.23_NCDZT15M3-OC/2018|EPI1823296|H5N6|2.3.4.4h
A/duck/China/FJ1923/2019|EPI1932009|H5N6|2.3.4.4h
A/duck/Fujian/10.23_FZHX0142-C/2018|EPI1823226|H5N6|
A/Duck/China/B5_NA/2019|EPI1890314|H5N6|
A/Duck/China/B8_NA/2019|EPI1890317|H5N6|
A/duck/China/FJ1931/2019|EPI1932011|H5N6|2.3.4.4h
A/Duck/China/B9_NA/2019|EPI1890318|H5N6|
A/Muscovy_duck/Vietnam/HN6606/2020|EPI1974311|H5N6|2.3.4.4h
A/chicken/Hunan/7.26_YYGK56V3-OC/2018|EPI1823318|H5N6|2.3.4.4h
A/chicken/Hunan/10.27_YYGK55B4-OC/2018|EPI1823322|H5N6|
A/goose/Fujian/3.15_FZHX0006-O/2018|EPI1822936|H5N6|
A/duck/Hunan/06.22_YYGK90T3-OC/2018|EPI1823315|H5N6|2.3.4.4h
A/goose/HeBei/GD07/2019|EPI1897236|H5N6|
A/chicken/Jiangxi/4.25_NCDZT70Q3-OC/2018|EPI1823303|H5N6|2.3.4.4h
A/chicken/Shanxi/2.22_TGRL001-O/2019|EPI1823383|H5N6|
A/chicken/Shandong/2.25_TAWL003-O/2019|EPI1823388|H5N6|2.3.4.4h
A/goose/Fujian/3.15_FZHX0009-C/2018|EPI1822903|H9N6|
A/chicken/Xinjiang/12.24_WLMQXL024-O/2018|EPI1823218|H6N6|
A/Whooper_swan/Xinjiang/6/2020|EPI1718981|H5N6|2.3.4.4h
A/chicken/Jiangxi/3.23_NCDZT57M3-OC/2018|EPI1823297|H5N6|2.3.4.4h
A/chicken/Anhui/8.28_YHZGS012-O/2018|EPI1823002|H5N6|2.3.4.2
A/chicken/Hunan/07.26_YYGK22V3-OC/2018|EPI1823316|H5N6|
A/chicken/Shandong/01.26_TAWL003-C/2019|EPI1823179|H5N6|
A/chicken/Shandong/12.26_TAWL015-O/2018|EPI1822948|H5N6|2.3.4.4h
A/chicken/Shanxi/8.28_TGRL020-O/2018|EPI1823130|H5N6|
A/chicken/Anhui/11.29_YHZGS019-O/2018|EPI1822975|H5N6|2.3.4.4h
A/chicken/Shanxi/12.01_TGRL025-C/2018|EPI1822957|H5N6|2.3.4.4h
A/chicken/Chongqing/10.28_RCTYH007-O/2018|EPI1822740|H5N2|2.3.4.4h
A/environment/Henan/11.29_XXHM003-E/2018|EPI1822995|H5N6|2.3.4.4h
A/Jiangsu/1/2018|EPI1342192|H5N6|2.3.4.4h
A/Jiangsu/1/2018|EPI1326833|H5N6|2.3.4.4h
A/China/Original/2018|EPI1508810|H5N6|2.3.4.4h
A/Jiangsu/32888/2018|EPI1352820|H5N6|2.3.4.4h
A/duck/Laos/NL-2175410/2021|EPI1921353|H5N6|2.3.4.4h
A/duck/Laos/NL-2175412/2021|EPI1921365|H5N6|2.3.4.4h
A/duck/Laos/NL-2175411/2021|EPI1921359|H5N6|2.3.4.4h
A/chicken/Laos/WKU-716/2021|EPI2774989|H5N6|2.3.4.4h
A/Chicken/Guangdong/211106-3/2021|EPI1942207|H5N6|2.3.4.4b
A/Goose/Guangdong/211106-2/2021|EPI1930185|H5N6|2.3.4.4b
A/Guangdong/lgf/2021|EPI2255269|H5N6|2.3.4.4b
A/Guangdong/1/2021|EPI2200690|H5N6|2.3.4.4b
A/Goose/Guangdong/211057/2021|EPI1930332|H5N6|2.3.4.4b
A/goose/Guangdong/S4751/2021|EPI1997233|H5N6|2.3.4.4b
A/Changsha/1/2022|EPI2287050|H5N6|2.3.4.4b
A/duck/Hunan/S40199/2021|EPI1997201|H5N6|2.3.4.4b
23-JBN-F12-36_H5N6_segment6_NA
[Isolated virus in this study]
A/peregrine_falcon/Saga/4112A002/2023|EPI2898979|H5N6|2.3.4.4b
NA
0.05
Country
China
Bangladesh
Vietnam
South_Korea
Mongolia
Laos
Japan
Israel
Indonesia
Cambodia
Georgia
Subtype
H3N6
H4N6
H5N2
H5N6
H6N6
H7N6
H9N6
H10N6

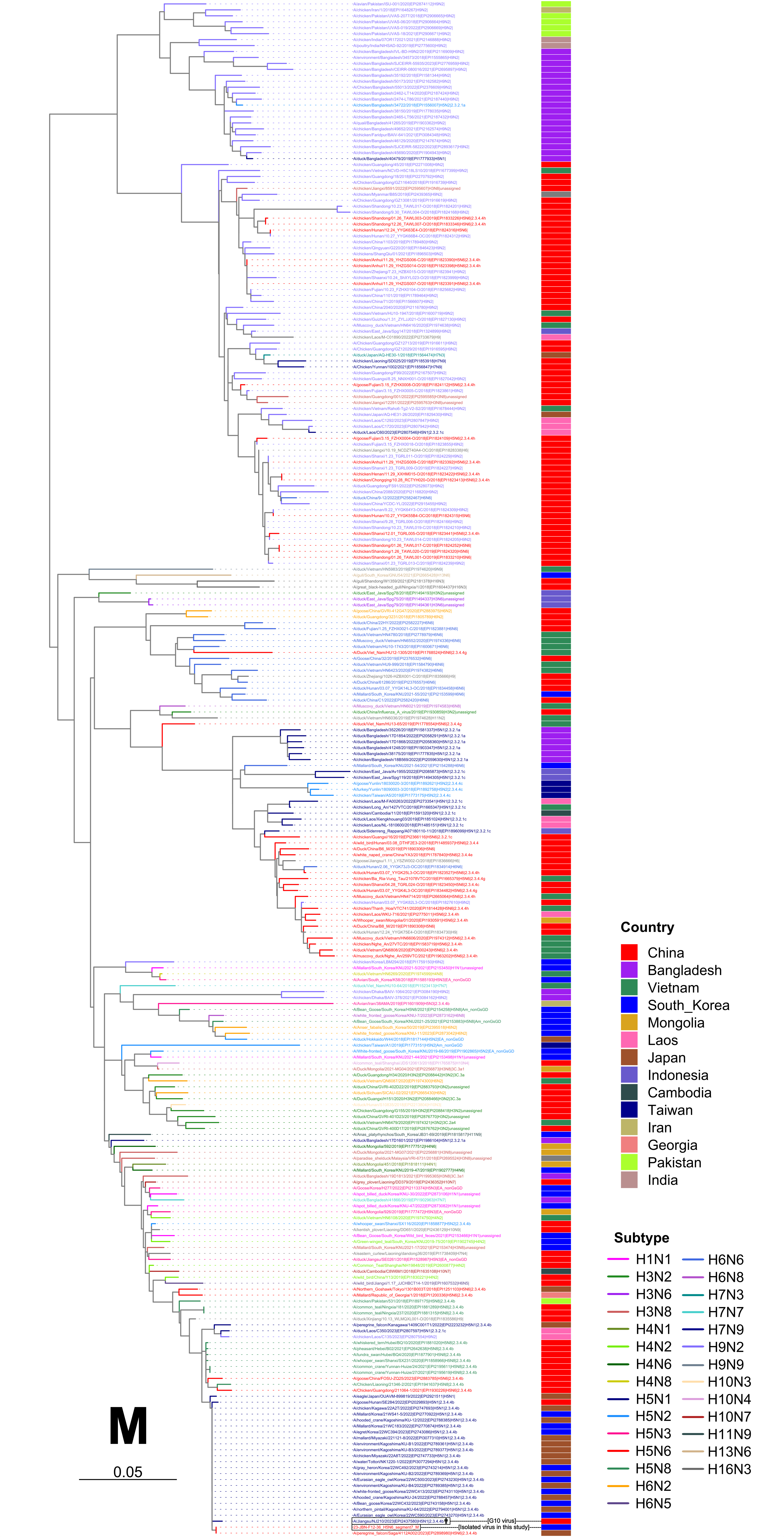

M
0.05
[G10 virus]
[Isolated virus in this study]
Country
China
Bangladesh
Vietnam
South_Korea
Mongolia
Laos
Japan
Indonesia
Cambodia
Taiwan
Iran
Georgia
Pakistan
India
Subtype
H1N1
H3N2
H3N6
H3N8
H4N1
H4N2
H4N6
H4N8
H5N1
H5N2
H5N3
H5N6
H5N8
H6N2
H6N5
H6N6
H6N8
H7N3
H7N7
H7N9
H9N2
H9N9
H10N3
H10N4
H10N7
H11N9
H13N6
H16N3

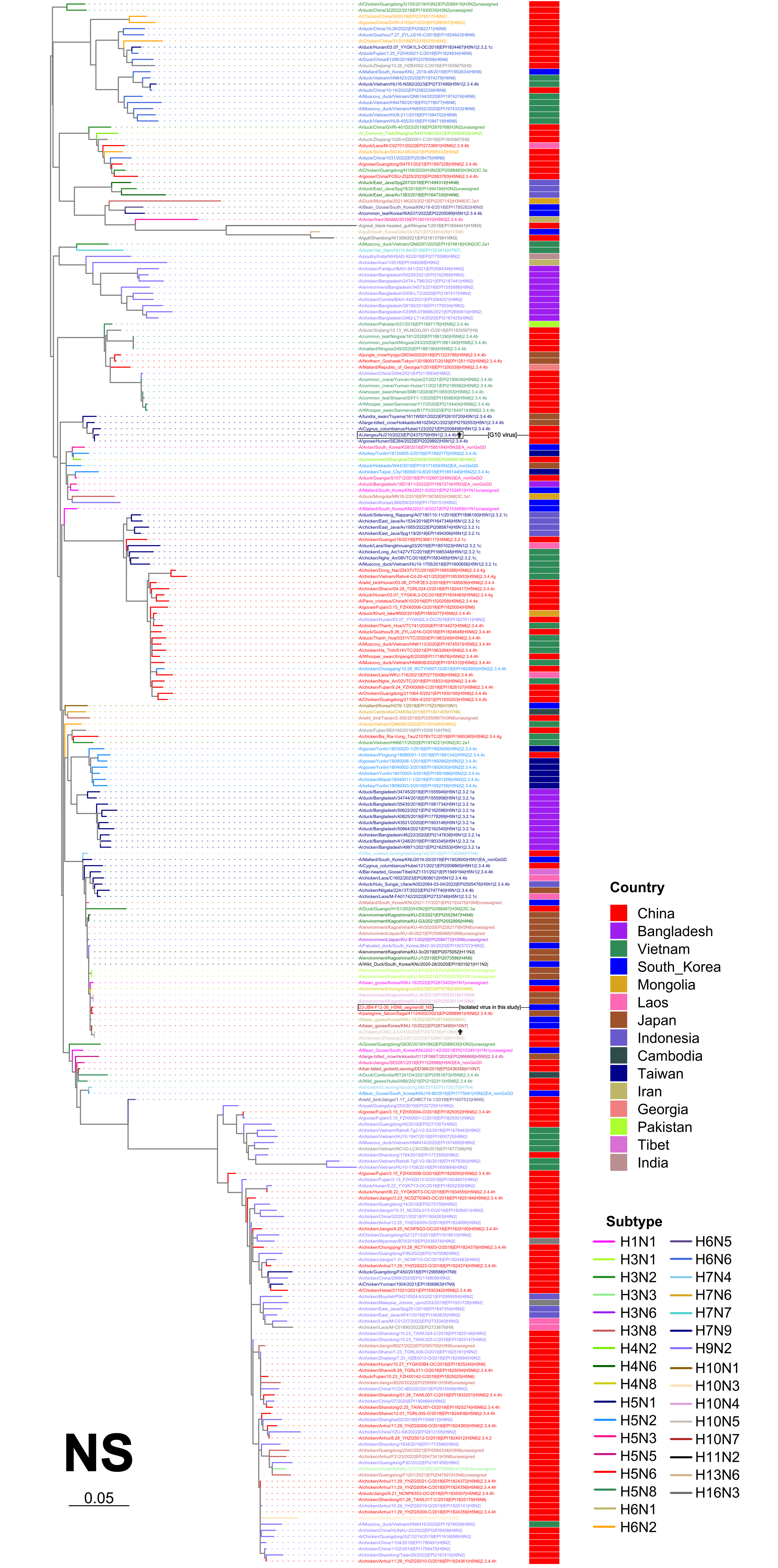

[G10 virus]
[Isolated virus in this study]
23-JBN-F12-36_H5N6_segment8_NS
NS
0.05
Country
China
Bangladesh
Vietnam
South_Korea
Mongolia
Laos
Japan
Indonesia
Cambodia
Taiwan
Iran
Georgia
Pakistan
Tibet
India
Subtype
H1N1
H3N1
H3N2
H3N3
H3N6
H3N8
H4N2
H4N6
H4N8
H5N1
H5N2
H5N3
H5N5
H5N6
H5N8
H6N1
H6N2
H6N5
H6N6
H7N4
H7N6
H7N7
H7N9
H9N2
H10N1
H10N3
H10N4
H10N5
H10N7
H11N2
H13N6
H16N3
